# The application of the loop-mediated isothermal amplification method for rapid detection of methicillin-resistant *Staphylococcus aureus*

**DOI:** 10.1016/j.nmni.2022.100960

**Published:** 2022-01-23

**Authors:** A.D. Khosravi, S. Khoshnood, E. Abbasi Montazeri, N. Jomehzadeh, M. Moradi, F. Shahi

**Affiliations:** 1) Infectious and Tropical Diseases Research Center, Health Research Institute, Ahvaz Jundishapur University of Medical Sciences, Ahvaz, Iran; 2) Department of Microbiology, School of Medicine, Ahvaz Jundishapur University of Medical Sciences, Ahvaz, Iran; 3) Clinical Microbiology Research Center, Ilam University of Medical Sciences, Ilam, Iran; 4) Department of Microbiology, Abadan Faculty of Medical Sciences, Abadan, Iran

**Keywords:** LAMP, mecA gene, MRSA, PCR, Rapid detection

## Abstract

Methicillin-resistant *Staphylococcus aureus* (MRSA) is an important problem associated with significant mortality and morbidity and well known as a predominant bacterial pathogen. The aim of this study was to identify MRSA strains. In this study (June 2018 to June 2019) isolates of *S. aureus* were obtained from patients referred to teaching hospitals of Ahvaz, Iran. All isolates were confirmed by conventional microbiological methods. In following, antimicrobial susceptibility testing (AST), MRSA screening, PCR detection of MRSA and LAMP assay were performed. Out of a total of 156 staphylococcal isolates, 126 isolates were identified as MRSA. Seventy-two (57.1%) MRSA isolates were recovered from wound. All MRSA isolates were sensitive to vancomycin, linezolid, teicoplanin, quinupristin-dalfopristin, and tigecycline. The results of LAMP showed 100% agreement with PCR. Sensitivity and specificity of the LAMP assays for the *mecA* genes were 100% and 100%, respectively. The LAMP assay is a rapid and simple method for the identifications of MRSA. Because of its performance without the need for specific instrumentation, this method can be easily employed in medical centers for the detection of *mecA*.

## Introduction

Due to the wide use of antibiotics, more antibiotic-resistant bacteria such as methicillin-resistant *Staphylococcus aureus* (MRSA) emerged, and the problem of antibiotic resistance became an important challenge throughout the world. MRSA is an important human pathogen associated with healthcare and community-acquired infections; including skin abscesses, necrotizing pneumonia, endocarditis, and joint infections [1, 2]. In recent decades, MRSA was reported from several countries and now is endemic in various hospitals worldwide, mainly in developing countries [3]. The main cause of resistance in MRSA is the production of penicillin-binding protein 2a (PBP2a), encoded by *mecA* gene and located on staphylococcal cassette chromosome mec (SCCmec), which has low affinity to β-lactam antibiotics [4].

Rapid detection of MRSA is crucial for the early treatment of patients and performance of infection control approaches to prevent its further outbreaks. There are various phenotypic methods for the examination of methicillin resistance in Staphylococci. However, these conventional methods are time-consuming and have a turnaround time of 18–24 h for diagnosis of MRSA [5].

Molecular biology-based methods such as DNA hybridization, polymerase chain reaction (PCR), and real-time PCR are currently used to identify the existence of MRSA directly from both environmental and clinical specimens. Although these techniques can detect the low number of bacterial cells, they needed special expensive equipment managed by trained personnel. Natomy et al. introduced a new nucleic acid amplification method named Loop-mediated isothermal amplification (LAMP) which is often used as an alternative to PCR-based methodologies in pathogen detection [6].

The LAMP assay relies on an auto-cycling strand displacement DNA synthesis performed by the *Bst* DNA polymerase enzyme under isothermal condition ranging from 60 °C to 65 °C and a set of 4–6 specially designed oligonucleotide primers. In this method, there is a possibility of separation of two strands of DNA and duplication simultaneously without the need temperature cycles and thermocycler devices using inexpensive equipment such as a hot water bath or a thermal block [7,8]. By now, the LAMP assay has been developed to rapid detection a variety of bacterial pathogens such as *Escherichia coli*, *Salmonella typhi*, *Leptospira species*, *Listeria monocytogenes, Vibrio parahaemolyticus Campylobacter jejuni* and *Shigella* species*.* Moreover, the LAMP technique was also indicated to be a useful diagnostic tool for the molecular detection of various *Staphylococcus* strains, and in particular methicillin-resistant *Staphylococcus aureus* [9,10].

The several advantages of the LAMP compared to the PCR test, including the simplicity, rapid response, high sensitivity and specificity. It could be suitable for onsite diagnosis of home-nursing patients or bedside diagnosis of hospitalized patients [11]. The aim of this study was to identify MRSA strains in patients referred to some educational hospitals in Ahvaz, Iran using LAMP and PCR methods.

## Material and methods

### Strains collection

In this cross-sectional study, (ethics number:IR.AJUMS.REC.1396.384) clinical isolates of *S. aureus* were obtained from patients referring to teaching hospital of Ahvaz, Iran during June 2018 to June 2019. Isolates were incubated at 37°C for 24 h on blood agar. Single colonies were identified with gram stain, catalase, oxidase, tube coagulase, DNase test, and growth on Mannitol salt agar (MSA) [12].

### Antimicrobial susceptibility testing

Antimicrobial susceptibility testing (AST) was performed by Kirby-Bauer disk diffusion method on Mueller–Hinton agar (MHA) according to Clinical and Laboratory Standards Institute (CLSI) guidelines [13] for antibiotics including: penicillin G (1unit), amoxicillin–clavulanic acid (30 μg), oxacillin (1 μg), azithromycin (15 μg), erythromycin (15 μg), cefazolin (30 μg), ceftazidime (30 μg), cefoxitin (30 μg), cefixim (5 μg), ceftriaxone (30 μg), ciprofloxacin (5 μg), trimethoprim–sulfamethoxazole (1,25/23,75 μg), gentamicin (10 μg), tobramycin (10 μg), doxycycline (30 μg), imipenem (10 μg), clindamycin (2 μg), vancomycin (30 μg), chloramphenicol (10 μg), linezolid (30 μg), teicoplanin (30 μg), quinupristin–dalfopristin (15 μg) and rifampicin (5 μg) (Mast, UK). The growth suspension for AST was prepared in 5 mL normal saline solution and the turbidity was adjusted to match that of 0.5 McFarland standards. Antibiotic discs were placed after 15 min of inoculation to MHA seeded with each isolate and were incubated for 18–24 h at 35–37 °C. The diameter of the zone of inhibition around the disc was measured. For accuracy, during the antibiotic screens, three independent replicates were performed. Multidrug resistance (MDR) was defined as resistance to three or more unique antibiotic classes in addition to beta-lactams [14].

### Methicillin-resistant *S. aureus* screening

Detection of MRSA was done using two methods according to the CLSI guidelines. First, inhibition zone less than or equal to 23 mm on Mueller Hinton Agar (MHA) with 30 μg cefoxitin disc. Second, inhibition zone on MHA containing 4% NaCl with oxacillin disc (1 μg) less than or equal to 10 mm [13].

### PCR detection of MRSA

Bacterial DNA extraction was performed in accordance with the boiling method. A volume of 2 μL of extracted DNA (50 ng) was added to a final volume of 20 μL PCR mixture containing 10 μL of Master Mix (Ampliqon, Denmark), 0.7 μL of 0.8 μmol/L each primer and 12.6 μL of sterile distilled water. The thermal cycling protocol for PCR was comprised 95 °C for 3 min, followed by 35 cycles of 94 °C for 1 min, 53 °C for 30 s and 72 °C for 1 min, with a final extension at 72 °C for 6 min. The amplified products were visualized by electrophoresis in 2% agarose gels stained with ethidium bromide.

### Primer design for LAMP assay

The sequences of the *mecA* gene of *S. aureus* with gene ID KC243783.1 was downloaded from the National Center for Biotechnology Information (NCBI) GenBank database. Then, LAMP primers were designed using the Primer Explorer V5 software (http://primerexplorer.jp/). A set of primers including FIP, BIP, F3 and B3 are shown in Table 1.

**Table 1:** Primers used in LAMP and PCR

Method	Target	Sequence(5'–>3′)
LAMP	*mecA*	F3: AGAAAAAGCGACTTCACATC
		B3: GCCATCTTTTTTCTTTTTCTCT
		FIP: TCCCTTTTTACCAATAACTGCATCATTATGTTGGTCCCATTAACTCT
		BIP: AAGCTCCAACATGAAGATGGCCGATTGTATTGCTATTATCGTCAA
PCR	*mecA*	F: ACGGTAACATTGATCGCAACG
		R: GGCCAATTCCACATTGTTTCG

### Standardization of the LAMP reactions

As mentioned above, bacterial DNA extraction was performed in accordance with the boiling method. Optimization of the LAMP assay was done MRSA NCTC 10442 in a 20 μL reaction mixture containing different concentration of each F3, B3, FIP and BIP primers, 8 U of large fragment *Bst* DNA polymerase (New England Biolabs, UK), 2.5 μL 10 × *Bst* buffer, different concentrations of MgSO_4_ (2, 4, 6, 8, and 10 mM)_,_ different concentration (4, 6, 8, and 10 mM) of dNTPs and 100 ng of DNA template. The mixture was incubated at 60 °C–65 °C for 60 and 90 min. Followed by enzyme inactivation at 90 °C for 5 min. LAMP products were analyzed by 2% agarose gel electrophoresis.

### Specificity and sensitivity determination of LAMP assays

The specificity of LAMP assay was assessed using MSSA ATCC12598 and MRSA NCTC 10442. Also, the detection limit of PCR and LAMP reactions determined using serially diluted DNA templates of MRSA 10442 strain. Then, all products was analyzed by methods mentioned above.

### Evaluation of the LAMP assay on bacterial cultures

After LAMP optimization, it carried out on all clinical *Staphylococcus* strains. Then amplicons were evaluated by adding 1 μL SYBR Green® I (Thermo Fisher Scientific) 0.1% to each reaction tube. The color changing from orange to green under UV was considered as positive. The experiment was done at least three times for each strain and the LAMP results were compared with PCR method.

### Statistical analysis

Descriptive data were analyzed using Microsoft Excel and SPSS version 22 statistics software. Comparisons between culture, PCR, and LAMP were performed with χ2 using Fisher's exact test. In addition, *P*-value < 0.05 was considered as significance level.

## Results

### Bacterial isolates

**Fig. 1:**
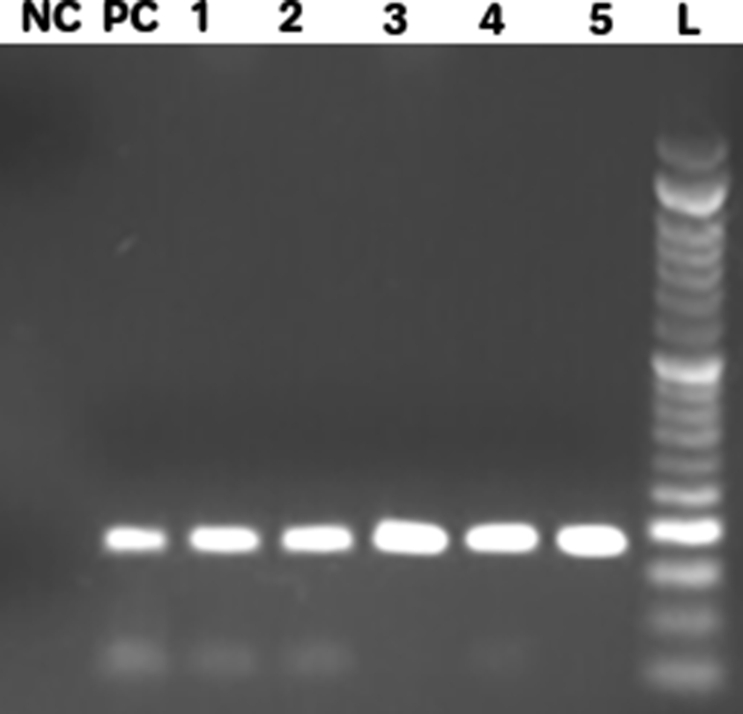
Electrophoresis of mecA gene PCR products. L: ladder, NC: Negative control (distilled water), PC: Positive control *S. aureus* ATCC25923, 1 to 5: Positive sample.

During 12 months, a total of 156 *S. aureus* isolates were characterized using standard microbiological tests. The age of the patients was 10–84 years (average 47 years), including 64 females and 92 males. Based on oxacillin and cefoxitin disk diffusion, oxacillin–salt agar screening and *mecA* gene PCR results 126 (80.8%) of *S. aureus* isolates were identified as MRSA. The PCR results are showed in Fig. 1. Seventy-two (57.1%) MRSA isolates were recovered from wound and other isolates were obtained from specimens, including, urine (35.7%, *n* = 45) endotracheal secretion (4.8%, *n* = 6), blood (2.4%, *n* = 3). Sources of MRSA and MSSA isolates according to the ward are presented in Table 2.

**Table 2:** Sources of MRSA and MSSA isolates according to ward

Ward	MSSA N (%)	MRSA N (%)	Total N (%)
Pediatric	3 (33.3)	6 (66.7)	9 (5.7)
General surgery	4 (19)	17 (81)	21 (13.5)
Internal women	10 (35.7)	18 (64.3)	28 (17.9)
Internal men	3 (21.4)	11 (78.6)	14 (9)
Outpatient department	2 (33.3)	4 (66.7)	6 (3.4)
Intensive care unit	2 (3.3)	59 (96.7)	61 (39.1)
Plastic surgery	6 (35.3)	11 (64.7)	17 (10.9)
Total	30 (19.2)	126(80.8%)	156 (100)

### Antibiotic resistance pattern of MRSA isolates

**Fig. 2:**
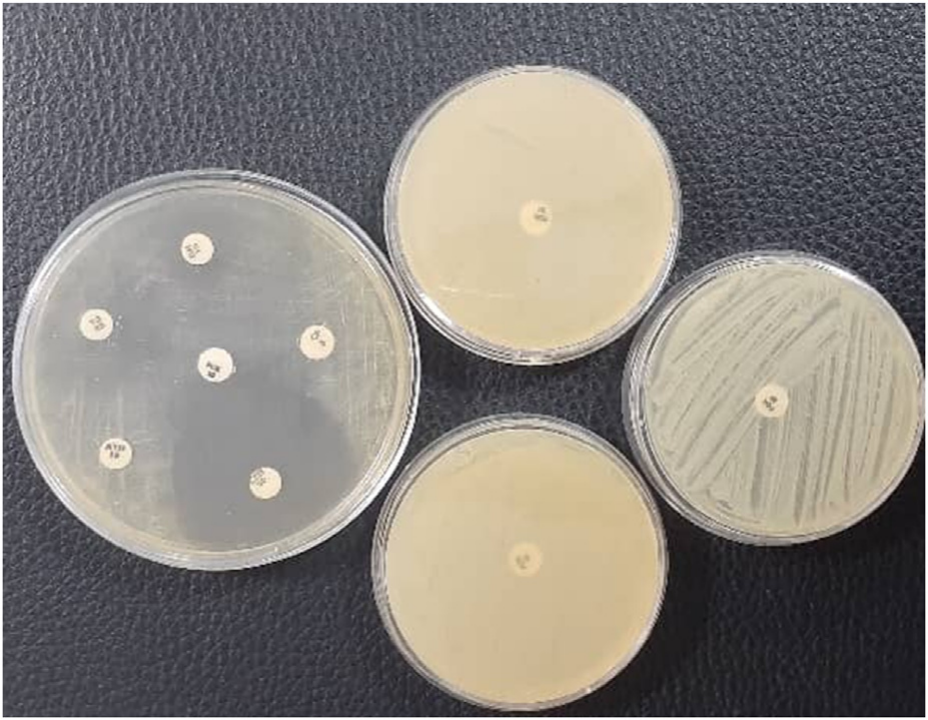
Result of disk diffusion method.

It was found that 100% of the MRSA isolates were sensitive to vancomycin, linezolid, teicoplanin, quinupristin–dalfopristin, and tigecycline while all isolates were resistant to amoxicillin–clavulanic acid and penicillin (Fig. 2). Also, more than 91% of isolates was resistant to azithromycin, erythromycin, ceftazidime, trimethoprim-sulfamethoxazole, and Imipenem. The antibiotic resistance pattern of MRSA isolates against all tested antibiotics is indicated in Table 3 with eight diverse patterns (Table 4). Most isolates (54.5%) were belonged to profile of number I.

**Table 3:** Antibiotic resistance pattern of MRSA isolates

Antibiotics	Resistant N (%)	Intermediate N (%)	Susceptible N (%)
Penicillin	156 (100)	0	0
Amoxicillin–clavulanic acid	156 (100)	0	0
Azithromycin	152 (97.4)	0	4 (2.6)
Erythromycin	154 (98.7)	0	2 (1.3)
Cefazolin	139 (89.1)	1 (0.6)	16 (11.3)
Ceftazidime	141 (90.4)	0	15 (9.6)
Ceftriaxone	16 (10.3)	2 (1.3)	138 (88.4)
Trimethoprim–sulfamethoxazole	141 (90.4)	0	15 (9.6)
Gentamicin	121 (77.6)	0	35 (22.4)
Tobramycin	85 (54.5)	0	71 (45.5)
Doxycycline	42 (27)	16 (10.3)	98 (62.7)
Imipenem	151 (96.8)	0	5 (3.2)
Clindamycin	151 (96.8)	0	5 (3.2)
Vancomycin	0	0	156 (100)
Linezolid	0	0	156 (100)
Teicoplanin	0	0	156 (100)
Quinupristin–dalfopristin	0	0	156 (100)
Tigecycline	0	0	156 (100)
Rifampicin	17 (10.9)	0	139 (89.1)

**Table 4:** Profiles of methicillin-resistant *Staphylococcus aureus* isolates

Multidrug-resistant profile	Phenotypic resistance	Number of isolates (%)
I	PEN-AMC-AZT-ERY-CZ-CAZ-SXT-GEN-TN-IMI-CLY	85 (54.5%)
II	PEN-AMC-AZT-ERY-CR0-DOX-IMI-CLY-RIF	15 (9.6%)
III	PEN-AMC-AZT-ERY-CZ-CAZ-SXT-GEN-DOX-IMI-CLY	26 (16.7%)
IV	PEN-AMC-AZT-ERY-CZ-CAZ-SXT-IMI-CLY	20 (12.8%)
V	PEN-AMC-AZT-ERY-CZ-CAZ-SXT-GEN-IMI-CLY	5 (3.2%)
VI	PEN-AMC-AZT-CAZ-CRO-ERY-GEN-AZT-CAZ-SXT-CZ-DOX	1 (0.6%)
VII	PEN-AMC-ERY-CZ-CAZ-SXT-GEN-RIF	2 (1.3%)
VIII	PEN-AMC-CAZ-GEN-SXT	2 (1.3%)

amoxicillin–clavulanic acid (AMC), azithromycin (AZT), cefazolin (CZ), ceftazidime (CAZ), ceftriaxone (CRO), clindamycin (CLY), doxycycline (DOX), erythromycin (ERY), gentamicin (GEN), imipenem (IMI), Penicillin G (PEN), rifampicin (RIF), trimethoprim–sulfamethoxazole (SXT), tobramycin (TN).

## LAMP

The optimized LAMP assay was performed thus: a 20 μL reaction mixture containing 0.2 pmol each of B3 and F3, 0.8 pmol each of BIP and FIP primers, 2.5 μL 10 × Bst buffer, 6 mM MgSO4, 1 μL Bst DNA polymerase (8 IU), 4 mM dNTPs, 1 μL (25 ng) DNA template, and 9 μL distilled deionized water. The optimized temperature was 63.5°C for 65 minutes.

**Fig. 3:**
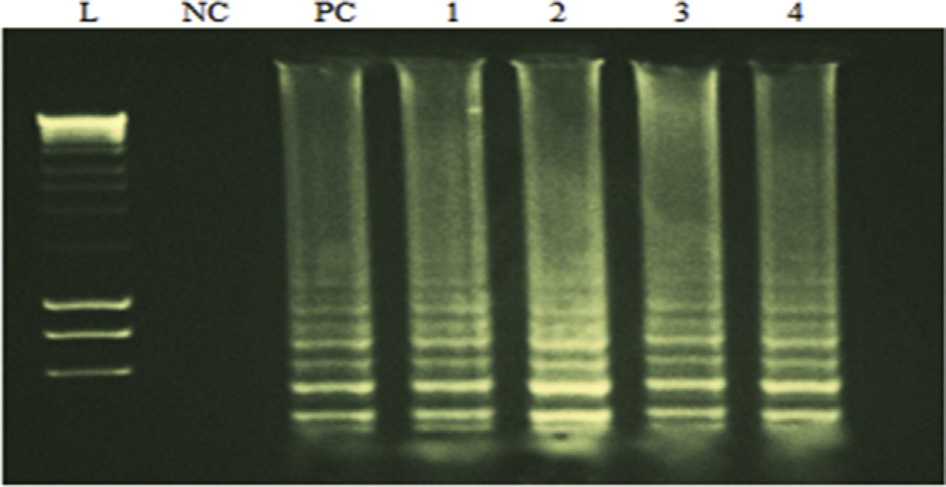
Electrophoresis of LAMP products. L: ladder, NC: Negative control (distilled water), PC: Positive control *S. aureus* ATCC25923, 1 to 4: Positive sample.

**Fig. 4:**
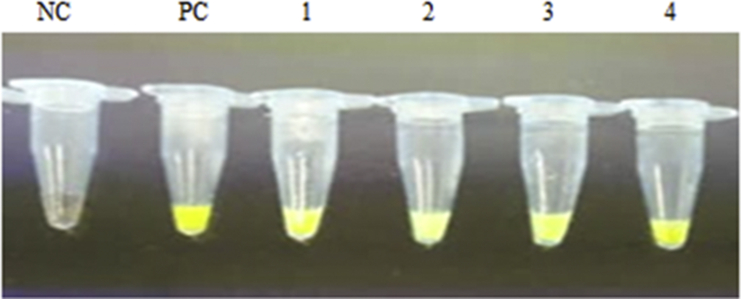
LAMP for detection of MRSA in clinical samples with SYBR green added. NC: Negative control (distilled water), PC: Positive control *S. aureus* ATCC25923, 1 to 4: Positive sample.

When the LAMP assay was used for detection of *mecA* gene in *S. aureus*, the results of LAMP showed 100% agreement with PCR. The results of electrophoresis of LAMP-amplification products are shown in Fig. 3. All clinical MSSA isolates (*n* = 30) and MSSA ATCC12598 were negative for *mecA.* All clinical MRSA isolates (*n* = 126) and MRSA NCTC 10442 were positive for *mecA* gene by PCR and LAMP assays (Fig. 4). Based on these data, sensitivity and specificity of the LAMP assays for the *mecA* genes were 100% and 100%, respectively.

## Discussion

MRSA is an important human pathogen responsible for various types of infections ranging from soft-tissue infections to bloodstream infections. Thus, accurate and rapid detection of MRSA is imperative for better implementation of infection control policies and effective treatment. The identification of MRSA by culture-based techniques often requires 1–2 days with the plating on blood agar and a series of biochemical tests. Of the nucleic acid amplification methods, PCR-based amplification methods have been developed for the diagnosis of MRSA [[15], [16], [17], [18], [19]].

Despite their simplicity and accuracy, these techniques are not widely used in private clinics as routine diagnostic tools, due to the need for special equipment such as thermal cycler, electrophoresis set, and gel documentation system [1,20,21]. The simple PCR methods usually include many steps for DNA extraction and long PCR protocols requiring several hours [22]. By using a multiplex PCR assay, investigators reduced the time for identification to approximately 2 h, but this reaction needed multiple primer sets to obtain the results [23]. Authors also proposed the use of real-time PCR, but this system is not accessible in many health care settings because it is more expensive than the conventional PCR-method [24,25]. However, the LAMP method requires only a conventional heating block.

Therefore, in the present study, we applied the LAMP method to detect MRSA. We generated specific primers *mecA* and analyzed all isolates. All MRSA isolates were detected by PCR as the reference. The detection results of the LAMP method were 100% mecA, which was 100% consistent with the PCR. Compared to the PCR, the entire process of the LAMP method needs less experimental condition requirements and its specificity and accuracy are equal to those of the PCR method. We have also demonstrated the LAMP methods for *mecA* with high specificity and sensitivity were accomplished within 1 hour. Also, under the constant temperature and by the naked eye examination, the LAMP obtained great advantages in rapidity and simplicity. A similar observation has been previously reported. Ting Lim *et al.*, reported that the sensitivity and specificity of the LAMP method were comparable to those of the PCR assay [26].

Based on their results, both LAMP and PCR assays showed 100% specificity, and the LAMP assay was approximately five times more sensitive than PCR assay. Likewise, subsequent investigation observed nearly the same percentages. Chen *et al.* showed that the diagnostic value of LAMP was identical with PCR and LAMP offers an alternative detection assay for *mecA* [1]. Similarly, according to the study conducted by Misawa *et al.*, the diagnostic values of LAMP, compared to a PCR assay, were 92.3% and 96.2% sensitivity and 100% and 100% specificity, respectively [27].

They indicated that the LAMP method is more cost-effective and provides excellent availability for rapid diagnosis in a clinical laboratory. Hanaki and colleagues showed that LAMP targeting the *mecA* gene associated with methicillin resistance identified MRSA with 100% specificity [28]. They also confirmed that the LAMP assays could be useful for the rapid identification of *S. aureus* isolates and determination of their antibiotic resistance patterns about methicillin. The results of a study performed by Koide *et al.*, show that LAMP, as an alternative technique to the PCR assays, is a powerful tool for the rapid identification of MRSA [7]. They conclude that through the naked eye inspection, the LAMP obtained great advantages in simplicity and rapidity. Also, it was accomplished in an hour with high specificity and sensitivity. LAMP assay is also useful for direct MRSA detection in clinical samples. According to Wang and others, the results of LAMP and PCR for diagnosis of MRSA in the clinical blood samples were the same as those of culture identification, which demonstrated that the LAMP assay can be used in the detection of clinical samples [2].

An important limitation of this study was that the *mecA*-LAMP assays alone may not discriminate between MRSA and coagulase-negative Staphylococci, because *mecA* is also widely distributed among these isolates. Thus, some modifications may be needed to apply this LAMP MRSA method, for the direct detection of MRSA from clinical samples.

## Conclusions

The LAMP assay is a rapid, flexible, and simple tool for the identifications of MRSA. Because of its performance without the need for specific instrumentation, this method can be easily used in any microbiology laboratories for the detection of *mecA*.

## Credit author statement

Azar Dokht Khosravi: Conceptualization, Funding acquisition, Project administration, Methodology. Saeed Khoshnood: Validation, Investigation, Visualization. Effat Abbasi Montazei: Resources, Supervision. Nabi Jomehzadeh: Formal analysis, Software. Melika Moradi: Investigation. Fatemeh Shahi: Data curation, Investigation, Methodology, Writing – original draft; Writing – review & editing, Validation.

## Transparency declaration

The authors declare no conflict of interest.
